# Kynurenine Pathway Metabolites as Biomarkers in Alzheimer's Disease

**DOI:** 10.1155/2022/9484217

**Published:** 2022-01-19

**Authors:** Yuqing Liang, Shan Xie, Yanyun He, Manru Xu, Xi Qiao, Yue Zhu, Wenbin Wu

**Affiliations:** Department of Geriatrics, Hospital of Chengdu University of Traditional Chinese Medicine, Chengdu, Sichuan 610072, China

## Abstract

Alzheimer's disease (AD) is a progressive neurodegenerative disorder that deteriorates cognitive function. Patients with AD generally exhibit neuroinflammation, elevated beta-amyloid (A*β*), *tau* phosphorylation (*p-tau*), and other pathological changes in the brain. The kynurenine pathway (KP) and several of its metabolites, especially quinolinic acid (QA), are considered to be involved in the neuropathogenesis of AD. The important metabolites and key enzymes show significant importance in neuroinflammation and AD. Meanwhile, the discovery of changed levels of KP metabolites in patients with AD suggests that KP metabolites may have a prominent role in the pathogenesis of AD. Further, some KP metabolites exhibit other effects on the brain, such as oxidative stress regulation and neurotoxicity. Both analogs of the neuroprotective and antineuroinflammation metabolites and small molecule enzyme inhibitors preventing the formation of neurotoxic and neuroinflammation compounds may have potential therapeutic significance. This review focused on the KP metabolites through the relationship of neuroinflammation in AD, significant KP metabolites, and associated molecular mechanisms as well as the utility of these metabolites as biomarkers and therapeutic targets for AD. The objective is to provide references to find biomarkers and therapeutic targets for patients with AD.

## 1. Introduction

Over 55 million people globally live with dementia, but only 25% are properly diagnosed since diagnostic criteria for dementia are unclear [1]. Alzheimer's disease (AD) is the main cause of dementia. The primary clinical manifestation of AD is cognitive impairment, characterized by a comprehensive decline in cognitive, behavioral, language, and other brain functions. Pathological manifestations of AD involve the extracellular deposition of beta-amyloid (A*β*), intracellular neurofibrillary tangles (NFT), *tau* phosphorylation (*p-tau*), and so on. Currently, the pathogenesis of AD remains unknown and many hypotheses are based on observed pathological changes (e.g., the A*β* waterfall hypothesis) [2, 3]. The discovery of inflammatory biomarkers, interleukin- (IL-) 1*β* [4, 5], and AD risk genes associated with innate immune function, apolipoprotein E (APOE) and triggering receptor expressed on myeloid cells 2 (TREM2), in AD patients suggests that neuroinflammation is essential in pathogenesis [2, 6].

The kynurenine pathway (KP) is crucial for peripheral and central catabolism of the essential amino acid, L-tryptophan (L-TRP). An imbalance in KP metabolism is a significant contributor to neuroinflammation. For example, overactivation of KP may result in excessive production of the excitotoxin quinolinic acid (QA) by activated microglia and perivascular and infiltrating macrophages [7–9]. A low level of cinnabarinic acid (CA) likely protects neurons but can be oxidized by 3-hydroxyanthranilic acid (3-HANA) to produce free radical superoxide anion and hydrogen peroxide [10, 11]. Conversely, picolinic acid (PA) has a dual function in immune regulation and can limit the excitatory toxicity of QA [12]. An interaction exists between KP metabolite imbalance and neuroinflammation. In a case-control study of clinical and biomarker-confirmed AD patients and cognitively healthy controls with paired plasma and cerebrospinal fluid (CSF) samples, metabolic profiling has been performed and the results showed that the specific association of amino acids and L-TRP catabolites with AD CSF biomarkers suggests a close relationship with core AD pathology [13]. And another metabolomic analysis of AD patients and healthy seniors suggests that the involvement of polyamine and L-TRP-KYN metabolisms was observed in the postmortem cerebrospinal fluid (PCSF) samples [14]. In addition, studies found that KP metabolites are associated with pathological changes and cognitive function in AD, such as QA [7–9, 15–25]. This work first discusses the mechanisms of AD neuroinflammation and KP regulation. Second, we summarized the relationship between neuroinflammation in AD and significant KP metabolites and molecular mechanisms. Ultimately, we discuss the applicability of KP metabolites as biomarkers for AD and provide references for the search for biomarkers and therapeutic targets.

## 2. Neuroinflammation in AD

Neuroinflammation is a response within the central nervous system caused by pathological damage, such as infection, trauma, ischemia, and toxin accumulation. This process involves the production of large quantities of proinflammatory cytokines, such as IL-1*β* and IL-6 and tumor necrosis factor- (TNF-) *α*; chemokine ligands, such as chemokine (C-C motif) ligand (CCL) 1 and CCL5; and small molecule messengers, such as prostaglandin, nitric oxide (NO), and reactive oxygen species (ROS) [26]. These mediators are produced by resident central nervous system (CNS) glia (microglia and astrocytes), endothelial cells, and peripherally derived immune cells [26–28]. Neuroinflammation is a critical pathophysiological feature of AD and may be a cause of the condition [29].

Microglial surface receptors recognize pathogen and paraprotein (e.g., A*β*) and induce microglial activation through endocytosis. Chemokine receptors and interferons are activated. These latter molecules are major components of the neuroinflammatory process [30, 31]. Reactive microglia are closely colocated with amyloid plaques in AD patients. These cells exhibit diverse phenotypes and interact with A*β* and *tau* types and with neuronal circuits [29, 32]. Microglia activated by A*β* produce the proinflammatory cytokines (such as IL-1*β*, IL-6, IL-8, and TNF), anti-inflammatory cytokine (transforming growth factor- (TGF-) *β*), chemokines (such as monocyte chemotactic protein-1 (MCP-1) and macrophage inflammatory protein-1*α* (MIP-1*α*)), cell adhesion molecules, NO, and ROS that lead to neurological dysfunction and death [33–36]. And the microglia receptors mainly include toll-like receptors (TLR), such as TLR1 and TLR2, and CD14, CD47, *α*6*β*1 integrin, and scavenger receptors. In turn, these receptors activate molecular pathways that induce microglial phenotypic changes [37–39]. For instance, TLR2 is a primary receptor for A*β* peptide to trigger neuroinflammatory activation, which can recognize and bind to A*β* and induce IL-8 and TNF expression [39]. The transition to disease-associated microglia is associated with downregulation of homeostatic genes and upregulation of genes with recognized association with AD, including APOE, TREM2, and TYRO protein tyrosine kinase-binding protein (TYROBP) [40]. Microglial activation depends largely on TREM2, and the role of TREM2 is important in the pathogenesis of AD [40–42]. The TREM2-TYROBP pathway is the main microglial signaling pathway and has been found to interact with both A*β*42 and *tau*-induced pathways at the gene expression level [43]. Several studies demonstrated that TREM signaling could suppress half of the pathways induced by A*β*, implying a defensive role for microglia, and in a *tau*-TREM2-TYROBP coexpression model, microglia acted synergistically to exacerbate *tau*-mediated neurodegeneration [44].

Microglia in aged brains show functional impairment and are prone to chronic activation that may contribute to AD [45, 46]. Microglial activation is a key element in promoting neuroinflammation and is closely related to pathological changes of AD [29]. Further, the peak in microglial activation may occur during the preclinical phase of AD, accompanied by A*β* deposition. A*β* clearance in later stages of AD decreases and *tau* accumulates. This condition compromises the defense function of microglia and triggers continuous and harmful microglial activation [29]. However, activation of microglia appears to inhibit progression in patients with mild cognitive impairment (MCI) [47]. These microglia may degrade and clear A*β* [30, 31, 33, 48–50]. Microglial responses to deferent pathological stimuli substantially alter AD progression. Effects mediated by microglia exhibit different effects along the disease trajectory depending on individual susceptibility and may be influenced by prior microglial priming [29].

Elevated proinflammatory cytokines are observed in the serum, brain, and CSF of AD patients [51, 52]. A significant correlation between levels of proinflammatory cytokines and cognitive decline is likely [53, 54]. Several meta-analyses and systematic reviews have described this relationship and suggest that AD is accompanied by peripheral and CNS-derived inflammatory processes [4, 5]. Zenaro et al. found that neutrophil infiltration in the CNS induces a neuroinflammatory cascade via LFA-1 integrin. This process exacerbates the pathological and cognitive decline in AD [55]. CXCL12 injection *in vivo* induces an increase in monocytes in the circulation of mice, and CXCL12 may promote migration of monocytes into the brain perivascular space and their transformation into PVMs. The latter action may be due to the upregulation of PECAM-1, which contributes to neuroinflammation and memory decline without crossing the blood-brain (BBB) or spinal cord barrier (SCB) [56]. Matrix metalloproteinases can aggravate inflammatory damage to the BBB by activating free radicals and proinflammatory cytokines [57–59]. Multiple microRNAs form positive and negative regulatory feedback loops with transcription factors, participate in neuroinflammatory responses caused by hypoxia/ischemia, and alter protein kinase activity and *tau* phosphorylation patterns in the brain via activating glial cells or affecting the expression of cytokines in neurons [60, 61]. Knocking out of Calhm2, a calcium homeostasis regulatory protein, in AD model mice significantly reduced inflammatory activation and increased the phagocytosis of microglia. A*β* deposition, neuroinflammation levels, and cognitive impairment of mice subsequently improved. Thus, neuroinflammation processes are closely related to Calhm2 regulation of calcium influx in microglia [62].

Activation of the inflammasome in the CNS is the key to establishing a chronic inflammatory environment that leads to neuronal dysfunction [29]. The recombinant NLR family, pyrin domain-containing protein 3 (NLRP3) inflammasome, in particular, is composed of nod-like receptor family members NLRP3, apoptosis-associated speck-like protein containing a CARD (ASC), and procaspase-1. This protein complex has become an important focus of research due to its central role in neuroinflammatory pathways [63]. NLRP3 recognizes danger signals of bacteria, viruses, and other endogenous factors and activates caspase-1 to induce the production of proinflammatory cytokines IL-1*β* and IL-18 [63, 64]. Microglia are critical for the initial appearance of A*β* plaques and the structure of the NLRP3 inflammasome. These cells complete the assembly of the NLRP3 inflammasome, leading to the formation of ASC that contributes to the deposition of plaques [65, 66]. A high concentration of A*β* can induce the expression of IL-1*β* and TNF-*α* in glial cells; A*β* deposition can also promote *tau* lesions by activating the NLRP3 inflammasome. This activation can also induce phosphorylation of *tau* accompanied by upregulation of inflammatory factors, such as IL-1*β* and IL-19 [64, 67]. A*β*1-42 peptide activates the TLR2, TLR4, and TLR6 to promote neuroinflammation [39]. The process continues with immune system activation and the production of inflammatory mediators [68]. Levels of *tau* phosphorylation decrease compared with the normal mice after injecting A*β* into the head of NLRP3- or ASC-deficient mice [67]. An NLRP3 inhibitor, OLT1177, reduces the activation of microglia and the formation of A*β* plaques in the cerebral cortex in an APP/PS1 mouse model of AD. These mice also showed less cognitive impairment [65, 69].

Overall, a correlation exists between neuroinflammation and AD; treatment for relevant markers of neuroinflammation can alleviate the progression of the disease.

## 3. The Kynurenine Pathway

L-TRP is an essential amino acid obtained from the diet. L-TRP is a cross-kingdom starting material for critical downstream metabolites involved in the synthesis of some indole-related compounds, such as 5-hydroxytryptophan (5-HT, serotonin), melatonin (MT), and kynurenine (KYN) [70–73]. L-TRP exists in unbound and bound forms in the blood in a ratio of about 9 to 1. A balance between these forms in the circulation is important even though a big difference exists in their relative concentrations. Only free L-TRP can be transported across the BBB [74].

**Figure 1:**
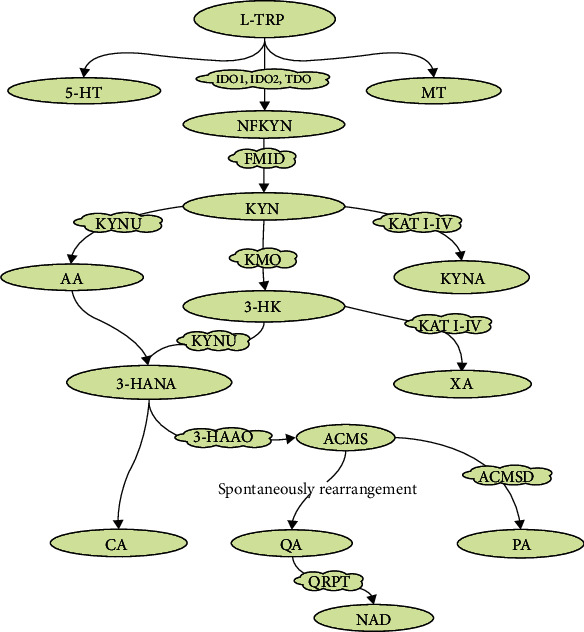
The kynurenine pathway of L-tryptophan metabolism. Green-filled ovals are the metabolic breakdown products in L-tryptophan metabolism, and cloud-like boxes filled with yellow are enzymes needed for oxidative and reductive reactions. L-TRP: L-tryptophan; 5-HT: 5-hydroxytryptophan; MT: melatonin; IDO: indoleamine-2,3-dioxygenase; TDO: tryptophan 2,3-dioxygenase; NFKYN: n-formyl-L-kynurenine; FMID: formamidase; KYN: kynurenine; KYNU: kynureninase; KMO: kynurenine 3-monooxygenase; KAT I-IV: kynurenine aminotransferase I-IV; AA: anthranilic acid; 3-HK: 3-hydroxy-L-kynurenine; KYNA: kynurenic acid; 3-HANA: 3-hydroxyanthranilic acid; XA: xanthurenic acid; 3-HAAO: 3-hydroxyanthranillate-3,4-dixogygenase; CA: cinnabarinic acid; ACMS: *α*-amino-*β*-carboxymuconate-*ε*-semialdehyde; ACMSD: ACMS decarboxylate; QA: quinolinic acid; PA: picolinic acid; QPRT: quinolone phosphoribosyl transferase; NAD: nicotinamide adenine dinucleotide.

The KP is central to peripheral and central catabolism of L-TRP (Figure 1). L-TRP concentrations in healthy adult human serum range from 1,000–50,000 ng/mL. The concentration of KYN is generally only one-tenth that of L-TRP [75, 76]. Only 2% of L-TRP is converted to 5-HT and MT that are critical for regulation of sleep, mood, appetite, and metabolism [71, 77]. Oxidative cleavage of the indole ring of L-TRP is followed by formation of N-formyl-L-kynurenine (NFKYN) by indoleamine-2,3-dioxygenase (IDO1 and IDO2) and tryptophan 2,3-dioxygenase (TDO) [78–80]. NFKYN is promptly degraded to KYN by kynurenine formamidase or kynurenine formylase [81, 82]. NFKYN is metabolized to KYN, the first stable intermediate metabolite, by formamidase. KYN is the central metabolite of the KP and is catabolized to anthranilic acid (AA), 3-hydroxy-L-kynurenine (3-HK), and kynurenic acid (KYNA) by kynureninase (KYNU), kynurenine 3-monooxygenase (KMO), and kynurenine aminotransferase I-IV (KAT I-IV), respectively [81–83]. 3-HK is converted to 3-hydroxyanthranilic acid (3-HANA), the precursor to the neurotoxic metabolite, QA [84, 85], and xanthurenic acid (XA) [86] by KYNU and KAT I-IV, respectively. 3-Hydroxyanthranillate-3,4-dixogygenase (3-HAAO) catalyzes the conversion of 3-HANA to QA [87, 88]. Also, a rapid reaction to an unstable intermediate product *α*-amino-*β*-carboxymuconate-*ε*-semialdehyde (ACMS) can be followed by spontaneous rearrangement to form QA [89]. ACMS is converted to PA by ACMS decarboxylate (ACMSD). PA can be produced in human glial cells and neurons and is neuroprotective. QA is a classic example of a biochemical double-edged sword, acting as both an essential metabolite and potent neurotoxin [90–92].

Further catabolism of 3-HANA can lead to the formation of CA. CA and XA are KYN metabolites generated by oxidative dimerization of 3-HANA and transamination of 3-hydroxy-L-kynurenine (3-HK), respectively [10]. Quinolone phosphoribosyl transferase (QPRT) metabolizes QA to NAD to facilitate energy production [90–93]. However, QPRT can slow the synthesis of NAD due to its limited enzyme capacity [90].

## 4. Metabolites and Molecular Mechanisms in the Kynurenine Pathway

### 4.1. Kynurenine

KYN is the first and main metabolite of L-TRP. The intermediate product, N-formylkynurenine, is produced by IDO1, IDO2, and TDO and subsequently converted to KYN by formamidase [78–80]. The physiological role of KYN is not well defined. An increase in KYN levels was found incidentally in peripheral and central cells during efforts to characterize tryptophan metabolism and the associated immune response [94]. KYN scavenges hydrogen peroxide and superoxide in specific pathways that produce ROS. Increased KYN levels also lead to a decrease in ROS production by activated neutrophils [95]. KYN is used in models of neurotoxicity as a neuroprotective agent, but observed effects are attributed to its metabolite, KYNA [96–98].

IDO1 and IDO2 catalyze the rate-limiting step in the formation of KYN, especially IDO1 [78–80]. IDO1 is also a pivotal enzyme in monocytes and macrophages in the immune system and microglia and astrocytes in the nervous system. *In vivo*, IDO1 activity is upregulated by inflammatory factors, such as lipopolysaccharide, interferon-*γ* (IFN-*γ*), immune response, and chronic stress, leading to an increase in KYN levels in the peripheral and central neural tissues [99, 100]. IFN-*γ* is notably effective for inducing the KP. IDO1 activity is regulated by TNF-*α*, IL-1*α*, TLR, pattern-related injury, or memory recognition cells in the immune system. These factors all increase NF-*κ*B signaling, which leads to further immunological dysregulation [101]. *γ*-[Glu]n-Trp (EW) decreases IDO activity by inhibiting inflammatory cytokine production (TNF-*α*, IL-6, IL-1*β*, and IFN-*γ*). This decrease reduces KP metabolism and restores normal behavior in mice with experimental depression [102].

#### 4.1.1. Kynurenine/L-Tryptophan

More than 90% of L-TRP in the periphery can be converted to KYN [77]. An increase in the kynurenine/L-tryptophan (K/T) ratio may reflect an increased risk of CNS diseases and might be a useful biomarker of KP metabolic disorders [103, 104]. That is, risk increases as more L-TRP is converted to KYN. Activation of IDO1 by inflammatory cytokines can increase the production of KYN and also lead to an increased K/T ratio [99, 100]. An increased peripheral K/T ratio implies an increase in the amount of KYN available for passive transport across the BBB [105, 106] thus raising the concentration of KYN in the brain.

Consequently, the K/T ratio is an important indicator of the formation of downstream metabolites in the KP. An increased K/T ratio is a prominent feature of inflammation and mental disorders in AD studies [107–109]. Further, IDO activity can be monitored by circulating K/T [110].

#### 4.1.2. KYN-AHR Signaling

AHR is closely associated with KYN level. AHR is a ligand-activated transcription factor and a node in the versatile intrakingdom communication system that binds molecules, such as L-TRP, with polyaromatic hydrocarbon rings [111, 112]. AHR is a highly conserved sensor for a wide variety of chemicals from environmental pollutants to constituents of food, cells, and microorganisms. Its role is fundamental to biological processes. Dysregulation of the AHR pathway is associated with a variety of maladies, including autoimmune diseases and cancer. AHR is thus a promising target for drug development and host-directed therapy [113].

KYN is an endogenous ligand, exogenous receptor, and transcription factor of AHR, and a combination of the two supports the immunomodulatory role of AHR [114]. KYN activates the AHR pathway and leads to an increase in the extent of cerebral infarction in mice with experimentally induced stroke [115]. AHR inhibits the destruction and senescence of bone marrow mesenchymal stem cells induced by KYN *in vitro*, but underlying mechanisms are not clear [116]. The KYN-AHR signaling pathway reverses the inflammatory response of dendritic cells after exposure to lipopolysaccharide (LPS) or IFN-*γ* [117, 118].

Enzymes involved in the synthesis of KYN are pivotal for KYN-AHR signaling. Inhibition of IDO1 with n-acetyl 5-hydroxytryptamine, a positive allosteric modulator of IDO1, can eliminate neuroinflammation in mice with experimental autoimmune encephalomyelitis (AE), a condition associated with IDO-AHR signaling [119]. In addition, inhibition of IDO1/TDO with RY103 *in vitro* reduced proliferation of glioma cells mediated by KYN-AHR signaling [120]. KYN-AHR signaling is important for regulating immunity, tumor, and neuroinflammation. Enzymes related to KYN play an important role in KYN/AHR signaling during neuroinflammation.

KYN is a critical KP metabolite for molecular metabolic changes upstream and downstream in the pathway. KYN levels are regulated by enzymes involved in responses to oxidative stress and inflammation (Table 1). Few studies are available to characterize relationships among KYN and neuroinflammation, enzymes, cytokines, and signaling pathways. Exploration of the role of KYN might be critical to understanding neuroinflammation and CNS dysfunction.

### 4.2. 3-Hydroxy-L-kynurenine

3-HK is an important intermediate of the main branch of the KP pathway. It is produced by conversion of KYN by KMO [81–83]. 3-HK displays physiological characteristics similar to KYN. Thus, elevated levels of 3-HK are associated with neuroinflammation [121, 122]. However, the role of 3-HK in neuropathology remains poorly understood due to the relatively low number of *in vivo* studies that directly examine its contribution to pathology.

3-HK exhibits both antioxidation and prooxidation properties, depending on concentration. Lower concentrations are associated with strong prooxidant activity with neuronal toxicity [96] and oxidation resistance in higher concentrations [123]. 3-HK might stimulate glutathione S-transferase, superoxide dismutase, and an important transcription nuclear factor, erythroid-derived 2-like 2 (Nrf2), that is important for antioxidant regulation [121]. Therefore, altering the concentration of 3-HK may modulate antioxidant systems and activate redox cell sensing mechanisms during inflammation.

The distribution of 3-HK is similar to that of KYN with higher concentrations in the cerebral cortex, striatum, and hippocampus [124]. Colín-González et al. injected 3-HK into a rat striatum and observed that oxidation of 3-HK decreased with increasing time and decreasing concentration. However, a high concentration is not completely beneficial for health [121]. High doses of 3-HK administered directly to mice produced neurotoxic effects, concentration-dependent cognitive impairment, and other neuropsychiatric symptoms [110, 125]. Further, 3-HK-mediated neurotoxicity is related to toxicity dependent on QA-N-methyl-D-aspartic acid (NMDA) receptor interaction [121, 126]. The 3-HK/QA and 3-HK/KA ratios are possible biomarkers for neuropathological effects of KP metabolism [110].

### 4.3. 3-Hydroxyanthranilic Acid

As a precursor of QA, 3-HANA is an important downstream metabolite in the KP produced by KYNU. 3-HANA regulates the release of inflammatory cytokines, counters lipid peroxidation, and lowers lipid levels in neural-immune cells [127]. 3-HANA levels vary among diseases for reasons that are not clear. For example, levels are lower in patients with stroke or after arterial surgery but higher in patients with Huntington's Disease (HD) or depression [128]. 3-HANA and 3-HK generate hydrogen peroxide and promote apoptosis in monocytes stimulated by IFN-*γ in vitro* and *in vivo*. Antioxidants reduce this apoptotic effect [129–131]. 3-HANA disrupted mitochondria-mediated energy metabolism unrelated to ROS formation. 3-HK acts as an anti-inflammatory and antioxidant agent in astrocytes and microglia (Table 1). This action reflects inhibition of apoptosis in monocytes stimulated by cytokines [132] and upregulation of the antioxidant enzyme heme oxidase-1 [132]. Further, increasing the level of 3-HANA reduces the activation of macrophage inflammasomes and decreases lipid levels in obese mice [133]. 3-HANA contributes to mitochondria-mediated energy metabolism unrelated to ROS formation *in vitro* and *in vivo* [134]. This factor also regulates the proliferation of T cells that induce inflammation in the AHR-NF-*κ*B signaling pathway in dendritic cells [135]. Finally, 3-HANA removes nitric oxide that can dilate blood vessels and prevent thrombosis [136].

### 4.4. Quinolinic acid

QA is a downstream metabolite formed from conversion of 3-HA to ACS by 3-HAAO followed by spontaneous rearrangement to QA [7]. QA is a potent neurotoxin and an essential amino acid. Microglia are responsible for QA production, and astrocytes and neurons must take up external QA to produce NAD [19, 137]. Neuroinflammation induces excess QA in activated microglia and infiltrated macrophages, which may destroy neurons, astrocytes, and oligodendrocytes [7–9]. QA exhibits neurotoxicity through at least three different mechanisms, including excitotoxicity by NMDA receptor (NMDAR) activation, formation of ROS, and destabilization of the cytoskeleton [138, 139]. Many NMDAR containing NR2A and NR2B subunits are distributed in the forebrain. QA shows a preference for binding with NMDAR, and neurons in this region are more likely to be damaged [23]. Moreover, QA can generate free radicals, increase ROS formation, and cause lipid peroxidation. These actions all increase oxidative stress [15], thereby further contributing to the pathogenesis of AD [140–143].

Neuropathy is accompanied by elevated levels of QA, suggesting that QA may be an important molecule in the pathogenesis of neurological diseases [144]. Structural and chemical changes in neurons after injection of QA into the brain and a QA-induced HD model have been established [84, 145, 146]. An acute QA infusion into the striatum of 30-day-old rats stimulated NFL hyperphosphorylation within 30 min. This effect was associated with cAMP-dependent protein kinase A and protein kinase Ca2+/calmodulin-dependent protein kinase II. Thus, both neuronal and astrocyte cytoskeletons are vulnerable to QA toxicity [139, 147]. Intracerebroventricular injections of QA induce epileptic signs in mice and cause axon sparing lesions on neurons [23]. QA was shown to be toxic via excitation to oligodendrocytes [16] and neurons [17, 18], induced inflammation in glial cells, and promoted the occurrence of neuroinflammation [21]. QA is closely associated with the formation of A*β* [19] and *p-tau* [20], which are related to immune cell response in the brain. A*β*1-42 directly stimulates the expression of IDO, thereby enhancing QA production *in vitro* and *in vivo* [24, 25]. QA is also observed to induce learning and memory deficits [20, 148]. A significant dose-dependent reduction in glutamine synthetase activity was found with QA treatment, showing that QA is an important factor for astroglial activation, dysregulation, and cell death with potential relevance to AD and other neuroinflammatory diseases [149]. Further, subsequent inhibition of the KP upstream of QA demonstrates that downregulating QA improves behavioral deficits in LPS hippocampal animals [125] (Table 1).

QA may also be associated with neuropsychiatric problems, possibly increasing the risk of poststroke depression [150] and increasing the risk of depressive symptoms [22]. Further, intracellular QA levels increase dramatically in response to immune stimulation (e.g., pokeweed mitogen (PWM)) in immune cells, such as dendritic cells and microglia [90], that produce less QA than macrophages [110]. Hence, QA and the KYNA/QA ratio (see Section 4.5) are both associated with immune and inflammatory responses and may be crucial therapeutic targets in degenerative diseases, such as AD.

### 4.5. Kynurenic acid

KYN can be converted to KYNA mediated by KAT I-IV. KYNA can only be produced by astrocytes by KAT II in the brain [98, 151, 152]. KYNA, in contrast to QA, is an antagonist at NMDA and glycine sites of this ionotropic receptor [7]. KYNA at higher concentrations modulates the production and release of glutamate. This activity is likely the primary main mechanism for KYNA-induced impairment of cognitive function [153–156]. KYNA is also an antagonist at the *α*7nicotinic acetylcholine receptor (*α*7nAChR) and the orphan G-protein coupled receptor 35 (GPR35) [23, 157]. Notably, KYNA might antagonize excitatory toxicity and oxidative stress caused by QA via downregulation of transcription factor, Nrf2 [158]. This metabolite also reverses cytoskeletal perturbations in striatal neurons that arise following QA-induced excitation [158]. At high concentrations, KA blocks convulsions induced by glutamate and QA [159]. Elevated levels of KA in the brain also lead to cognitive dysfunction in the medial prefrontal cortex (mPFC). Additionally, KAT II inhibition blocks the generation of KYNA and increases the release of glutamate in the mPFC. This inhibition ameliorates cognitive impairment caused by excessive levels of KYNA [160–162]. KAT II-knockout mice exhibit lower levels of KA in the brain and improved cognition compared to control mice [162]. KYNA levels in the brain increase after immune activation of KP. Impairment of working memory follows [163], which may be related to the accumulation of KYNA in the prefrontal cortex.

### 4.6. Xanthurenic acid, Picolinic acid, and Cinnabarinic acid

XA is produced mainly in astrocytes, catalyzed by KAT II [110]. ROS clearance and Fe ion coordination complex formation with XA and KYN affect neurodegenerative diseases, but understanding this correlation is still incomplete [164]. XA is an antioxidant [129] and acts as an agonist at metabotropic glutamate receptors (mGLUR) mGLUR2 and mGLUR3 [165]. However, no relevant study exists that examines XA expression and AD.

CA and PA are generated by 3-HANA via two pathways. CA is oxidized by 3-HANA to produce superoxide anion and hydrogen peroxide, which induces ROS formation and caspase activation, inhibits the mitochondrial respiratory chain, and promotes apoptosis [11]. CA is an agonist of mGLUR4 [10]. CA levels in the brain are typically low, which seems to protect neurons. These levels may increase after immune stimulation [10]. This response might modulate immune responses, such as binding to immune receptors, such as AHR. This binding may regulate T cells and reduce neuroinflammation [166] with therapeutic implications for AD. PA is an unstable intermediate of 3-HANA decomposition produced by ACMSD. The production of PA is positively correlated with ACMSD activity [22, 89]. Generally, the concentration of PA in the periphery is higher than that in the CNS; PA levels can peak in adulthood but decline over time [93]. PA may have a dual function of immune regulation that induces or suppresses the expression of immune cells, such as macrophage proteins MIP-1*α* and MIP-1*β*, and T cells [12, 167, 168]. High doses of PA are neurotoxic but can reduce the excitatory toxicity of QA [12]. Further, PA has an antimicrobial function [169] (Table 1).

## 5. KP Metabolites as Biomarkers in AD

Current diagnostic methods for dementia are notoriously complex, involving expensive PET or MRI scans or CSF tests. Resources for these diagnostics, for health professionals trained to provide them, are limited globally [1]. Metabolomics is an approach that complements hypothesis-driven techniques and targets. Specific factors, with known associations, such as molecular signals (e.g., APOE *ε*4), are controversial targets for AD patients [170, 171]. Many pathways—lipid, uric acid, and amino acid metabolism [172–176], immune and inflammatory responses [177], BBB dysfunction, and metal ion homeostasis—can modulate AD. AD, as a multifactorial neurodegenerative disease associated with aging, requires careful diagnosis to accurately distinguish it from normal aging. Therefore, finding biomarkers related to AD is critical for the prevention, diagnosis, and treatment of AD.

Serum, CSF, and imaging examinations are often used for the diagnosis of AD patients. However, in most animal studies, pathological sections of brain tissue and CSF are generally used to detect known pathological products and possible biomarkers. In 2018, biomarkers were grouped into A (amyloid), T (*p-tau*), and N (neurodegeneration, measured by total *tau*(*t-tau*) where applicable) [178]. Differences between KP peripheral and central metabolism reflect varied metabolite function in the pathway. Consequently, both serum and CSF were analyzed to find biomarkers to aid AD diagnosis.

A large-scale multicenter study in Europe assessed metabolic phenotypes of urine and serum from the Europe-wide AddNeuroMed/Dementia Case Register biobank of patients clinically diagnosed with AD and MCI. 5-HT (urine, serum), KYN (serum), KYNA (urine), L-TRP (urine, serum), and K/T ratio (urine) were significantly lower in AD patients than in controls. The bioavailability of neurotransmitters in AD may be altered. Further, the significant increase in the K/T ratio might be due to increased activity of IDO and more active metabolism of KP associated with systemic inflammation [179].

The levels of KYNA in the CSF of AD patients are significantly increased compared with normal controls [13, 107]. Increased KYNA concentrations in CSF in patients with AD are not seen in other neurodegenerative diseases, such as frontotemporal dementia (FTD), amyotrophic lateral sclerosis, and progressive supranuclear palsy. KYNA may be specifically elevated in AD [180].

KYN and 5-HT are metabolites of L-TRP, and KYN is the main product produced by activation of KP. Patients with normal cognition, MCI, and AD were assessed in a clinical study for KYN, L-TRP, and 5-HT in serum, A*β*, and *tau* and KYN in CSF. An inflammatory signal cascade may occur during AD, related to the increase in KYN [181]. Higher K/T ratios were associated with many inflammatory markers and lower functional independence and memory scores. A*β* and complement systems may be key contributing factors in this process [181].

Plasma concentrations of KYN, 3-HANA, XA, QA, and L-TRP were found to be lower in AD patients compared to controls [109]. Specially, elevated QA was associated with poor cognition in older AD patients [109]. Another study found that the plasma concentrations of L-TRP and KYNA decreased and QA increased in AD, which proved the activation of peripheral KP in this type of dementia [182]. Plasma KYN and PA in AD patients were inversely correlated with CSF *p-tau* and *t-tau*, respectively. Moreover, increased 3-HK/KYN ratios correlated with *t-tau* in CSF of AD patients [107]. Further, L-TRP catabolites, KYNA and QA, showed significantly higher concentrations in the CSF of AD patients. These higher concentrations were correlated, as were other L-TRP pathway intermediates, with either CSF A*β*1-42 or *tau* and *p-tau*-181 [13]. Concentrations of KYNA were reduced in the CSF of AD patients. Also, serum KYN and QA levels strongly correlated with their respective content in CSF, and KYN in serum was negatively correlated with AD disease severity. Also, it has been suggested that the development of inflammatory-mediated neuropathology is correlated with changes in the ratio of KYNA to QA rather than QA levels alone [183]. KYN metabolites accumulated similarly with aging in serum and CSF in AD patients and control subjects. In contrast, KYNA was significantly reduced in the CSF of AD patients. Age- and disease-specific changes in cerebral KP activity might contribute to reduced neurogenesis and increased excitotoxicity in neurodegenerative disease [184].

These findings support KP involvement in AD pathogenesis and support the concept of therapeutic modulation of KP for the treatment of AD. Further, KYN (plasma, serum, and CSF), KYNA (serum, urine, and CSF), L-TRP (plasma, serum, and urine), K/T ratio (plasma, serum, urine, and CSF), 3-HANA (plasma), XA (plasma), and QA (plasma, CSF) were found to be related to the severity of AD. Metabolites of KP, such as KYN and QA, may be useful biomarkers for the early diagnosis of AD.  Table 2 lists biomarker studies in biological fluids that suggest notable associations of KP metabolites with AD.

## 6. Therapeutic Considerations

NMDAR blockers, such as MK-801 and memantine, reduce the glutamate neurotoxicity [185]. Memantine, commonly used for the treatment of AD, acts as an NMDAR antagonist and reduces QA-induced hippocampal injury [186]. KP metabolism has an important influence on the occurrence of AD and may be an important target for the treatment of AD. Changes in KYNA and QA are associated with cognitive impairment in AD patients [182]. QA is neurotoxic, while KYNA may exhibit neuroprotective effects [187]. The systemic administration of 4-chlorokynurenine (4-Cl-KYN), an NMDAR, did prevent QA-induced neurotoxicity in the hippocampus of the rats [188]. 4-Cl-KYN can be transformed into 7-chlorokynurenic acid (4-Cl-3-HANA), and thus, it can inhibit 3-HANA oxygenase and QA synthesis [189]. Therefore, modification of the KP through pharmacological inhibition of the enzymes of QA synthesis is a rational approach via which to divert the KP metabolism toward the neuroprotective KYNA [190]. Also, upregulating KYNA synthesis and reducing KP metabolites formed by QA may be a useful therapeutic strategy.

Regulating KP activity may improve some symptoms of AD in animal models [3, 191–193]. Inhibition of KYN metabolism in the blood to increase KYN transport to the brain increases brain KYNA production. This action prevents synaptic loss and improves memory function in mice with experimental AD [192].

In addition, intervention at the level of KP-related enzymes may also be important for AD treatment. A*β*1-42 directly stimulates IDO expression, thereby enhancing QA production *in vitro* and *in vivo* [24, 25]. The IDO inhibitor, coptisine, ameliorated AD-like signs in a mouse model of AD. Coptisine inhibition of IDO decreases the activation of microglia and astrocytes, consequently improving neuronal loss and A*β* formation [194]. Cognition, anxiety, and depression improved in 3XTG-AD mice treated with the novel IDO inhibitor, DWG-1036 [195]. Similarly, TDO inhibition reduces neurodegeneration in animal models of AD [196]. TDO is highly expressed in the brain tissues of both AD model mice and AD patients, suggesting that TDO-mediated KP activation may be involved in the formation of neurofibrillary tangles and senile plaques [197]. In addition, treatment of APP/PS1 mice with a TDO inhibitor for 4 weeks prevented deficits in hippocampal-dependent memory function [198]. Finally, inhibition of KMO reduces synaptic loss in a mouse AD model. Treatment of AD mice significantly improved symptoms and delayed disease progression [192, 199]. Thus, KP metabolites and enzymes are clinically significant targets for the treatment of AD.

## 7. Concluding Remarks

L-TRP metabolism by the KP regulates inflammation, immunity, and tumor progression. KP signaling is the result of various metabolic influences, such as inflammatory factors, and the early discovery of metabolites can support the early diagnosis and treatment of AD. Firstly, studies suggested that peripheral inflammatory factors played a key role in the early stage of the blood-cerebrospinal fluid barrier [5, 26]. Therefore, we should take a holistic view of inflammation that peripheral inflammation was associated with neuroinflammation. Secondly, KP metabolites and molecular mechanisms are complex and most of them have yet to be explored. Different biomarkers are the most appropriate from the perspective of molecular mechanisms and existing biomarker exploration. KYN, 3-HANA, and CA have an antineuroinflammatory effect which can inhibit AD. It is found that KYNA can lead to cognitive impairment. 3-HK, QA, and PA have neurotoxicity and promote AD. QA is also supposed to be closely related to the formation of A*β* and *p-tau* and to promote neuroinflammation. According to existing studies, elevated QA promotes pathological changes and neuroinflammation in AD. However, KYN might be more advantageous as a biomarker in related studies. This may be because QA poorly diffuses across the BBB and mainly exists in the CNS, while KYN can cross the BBB through the large neutral amino acid transporter. We consider that KYN might be more advantageous as a biomarker, while QA might be more advantageous as a therapeutic target. Therefore, future studies should carefully explore the biological functions of various important KP metabolites. Further mechanistic studies are needed to link KP metabolites with AD. However, modulation of the KP at various points in the pathway is likely to be beneficial. KP metabolites, enzyme inhibitors, and neutralizing antibodies are future therapeutic strategies in neurological disease.

## Figures and Tables

**Table 1:** Summary of individual KP metabolites with known receptor targets, key biological functions, negative effects, and effects associated with AD (debated^∗^).

Metabolite	Receptors	Biological functions	Negative effects	Effects associated with AD	Ref.
KYN	AHR	Immunomodulation, anticancer, oxidative stress regulation, neuroprotection^∗^, anti-inflammatory	Unknown	Antineuroinflammatory	[78–80, 95, 99, 100, 119, 120]
3-HK	Unknown	Oxidative stress regulation	Neurotoxicity	Neurotoxicity	[96, 110, 125]
3-HANA	Unknown	Anti-inflammatory, oxidative stress regulation, lipid-decreasing, immunomodulation	Unknown	Antineuroinflammatory	[127, 129–135]
QA	NMDAR	Proconvulsant, prooxidant	Neurotoxicity, proneuroinflammatory prodepression	Formation of A*β* and p-tau, proneuroinflammatory neurotoxicity	[7–9, 15–23]
KYNA	AHR, NMDAR, *α*7nAChR	Antioxidant, immunomodulation, anticonvulsant, antineurotoxicity	Unknown	Cognitive disorder	[23, 153–159, 163]
XA	mGLUR2/3^∗^	Antioxidant	Unknown	Unknown	[129, 165]
PA	Unknown	Anticonvulsant, antimicrobial, immunomodulation	Neurotoxicity	Neurotoxicity	[12, 167–169]
CA	AHR, mGLUR4	Immunomodulation, antineuroinflammatory	Proapoptosis	Antineuroinflammatory	[10, 11, 166]

**Table 2:** Studies of biomarkers in biofluids and their association with KP metabolites in AD.

Biofluid biomarker	Metabolites	Observation	Ref.
Serum	17 metabolites	Decreased concentration of L-TRP, KYN, and KYNA	[179]
	L-TRP, KYN, and 5-HT	Increased concentration of K/T	[181]

Plasma	17 metabolites	Decreased concentration of KYN, 3-HANA, XA, QA, and L-TRP	[109]
	6 metabolites	Decreased concentration of L-TRP and KYNA; increased concentration of QA	[182]

Urine	8 metabolites	Decreased concentration of KYNA, L-TRP, and K/T	[179]

CSF	L-TRP	Increased concentration of KYNA	[13, 107, 180]
	L-TRP	Increased concentration of KYNA and QA	[13]

CSF and serum	L-TRP, KYN, and 5-HT	Increased concentration of KYN	[181]
